# Durable Complete Response to Pembrolizumab for Postoperative Nodal Recurrence of Lynch Syndrome–Associated Upper Tract Urothelial Carcinoma: A Case Report

**DOI:** 10.1002/iju5.70293

**Published:** 2026-11-02

**Authors:** Kaya Atagi, Shinkuro Yamamoto, Nobutaka Shimizu, Mitsuko Iguchi, Ryu Shigehisa, Tomoya Nao, Tsutomu Shimamoto, Hideo Fukuhara, Satoshi Fukata, Keiji Inoue

**Affiliations:** ^1^ Department of Urology, Kochi Medical School Kochi University Nankoku Japan; ^2^ Pelvic Floor Center, Kochi Medical School Kochi University Nankoku Japan; ^3^ Department of Diagnostic Pathology, Kochi Medical School Kochi University Nankoku Japan

**Keywords:** complete response, Lynch syndrome, microsatellite instability, pembrolizumab, upper tract urothelial carcinoma

## Abstract

**Introduction:**

Germline mutations in mismatch repair genes cause Lynch syndrome and are associated with upper tract urothelial carcinoma (UTUC). Tumors with deficient mismatch repair (dMMR) or microsatellite instability–high (MSI‐H) may respond favorably to immune checkpoint inhibitors.

**Case Presentation:**

A 54‐year‐old woman with Lynch syndrome and a germline MSH2 mutation developed right renal pelvic carcinoma. The tumor was diagnosed as a pT3 high‐grade urothelial carcinoma, and the patient underwent laparoscopic nephroureterectomy. She was subsequently administered adjuvant gemcitabine and carboplatin due to a contraindication for cisplatin. Due to nodal recurrence, pembrolizumab was initiated at 200 mg every 3 weeks, achieving a complete radiological and metabolic response after five cycles. The course was complicated by immune‐related adrenal insufficiency, which was managed with corticosteroid replacement therapy.

**Conclusion:**

Pembrolizumab may achieve a durable complete response in Lynch syndrome–associated UTUC. MSI screening should be considered in UTUC to guide decision‐making.

## Keynote Message

Pembrolizumab achieved a durable complete response in a case of postoperative recurrent Lynch syndrome–associated upper tract urothelial carcinoma. This case of early‐onset urothelial carcinoma underscores the importance of microsatellite instability testing and highlights the therapeutic relevance of immune checkpoint inhibition observed in deficient mismatch repair tumors.

**Abbreviations**
dMMR: deficient mismatch repair
FDG‐PET: fluorodeoxyglucose positron emission tomography
FDG‐PET/CT: ^18^F‐fluorodeoxyglucose positron emission tomography/computed tomography
MLH1: MutL homolog 1
MMR: mismatch repair
MSH2: MutS homolog 2
MSH6: MutS homolog 6
MSI‐H: microsatellite instability–high
PD‐1: programmed cell death protein 1
PET/CT: Positron emission tomography and computed tomography
RECIST: response evaluation criteria in solid tumors
UTUC: upper tract urothelial carcinoma

## 1 Introduction

Upper tract urothelial carcinoma (UTUC) is a relatively uncommon malignant tumor [1]. Caused by germline mutations in DNA mismatch repair (MMR) genes such as MSH2, Lynch syndrome is often associated with an increased risk of UTUC, endometrial cancers, and colorectal cancers [1, 2, 3, 4, 5]. With increased tumor immunogenicity and sensitivity to immune checkpoint inhibitors, tumors arising in Lynch syndrome often exhibit microsatellite instability–high (MSI‐H) and deficient mismatch repair (dMMR) [2, 6].

However, durable complete responses have been rarely reported after immune checkpoint inhibition in Lynch syndrome‐associated UTUC [7, 8]. Furthermore, despite the strong association between Lynch syndrome and UTUC, MSI testing is not routinely performed in UC [1, 2, 5]. In this report, the importance of molecular profiling and immunotherapy in hereditary UC is highlighted in a case of postoperative nodal recurrence of Lynch syndrome–associated UTUC. This case achieved a durable complete response with pembrolizumab.

## 2 Case Presentation

A 54‐year‐old woman presented with gross hematuria and a medical history that included several surgically treated multiple primary cancers, including cecal, gastric, endometrial, and ovarian. MSI testing performed after resection of the cecal cancer revealed MSI‐H status. Subsequent germline genetic testing identified a pathogenic *MSH2* mutation, leading to a diagnosis of Lynch syndrome.

Imaging performed 2 years later showed a 25‐mm hypermetabolic mass at the right renal pelvis–ureteral junction with associated dilatation of the renal pelvis and calyces, without distant metastasis (Figure 1a–c). Because the lesion was considered clinically suspicious for invasive UTUC and the contralateral kidney was functional, radical nephroureterectomy with bladder cuff excision was selected with curative intent. The patient underwent laparoscopic radical nephroureterectomy with bladder cuff excision. Pathological examination revealed high‐grade UC that invaded the peri‐pelvic fat (pT3) (Figure 2a,b). Immunohistochemistry findings were consistent with dMMR, revealing loss of MSH2 and MSH6 expression associated with preserved MLH1 and PMS2 (Figure 2c–f).

**FIGURE 1:**
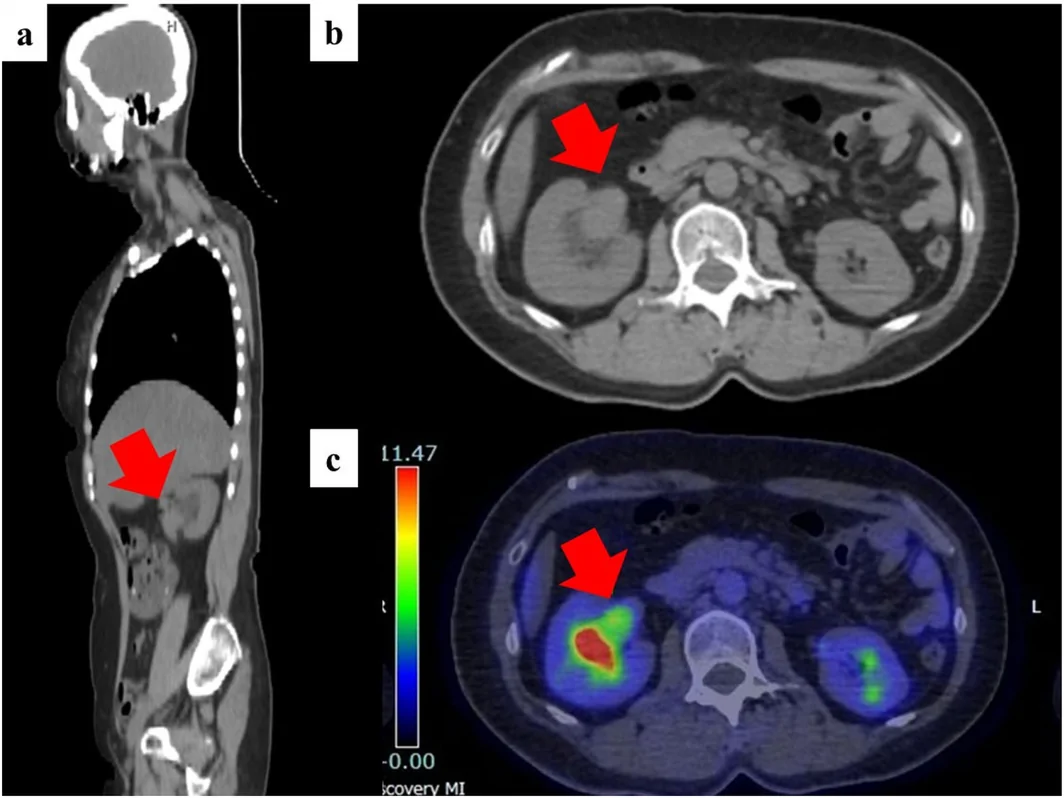
(a) Sagittal computed tomography (CT) image showing a 25‐mm soft‐tissue mass at the right renal pelvic–ureteral junction. (b) Axial CT image of the same lesion. (c) Fluorodeoxyglucose positron emission tomography image revealing intense uptake at the corresponding site (maximum standardized uptake value, SUVmax 8.4), which is associated with dilatation of the renal pelvis and calyces. No distant metastasis was observed.

**FIGURE 2:**
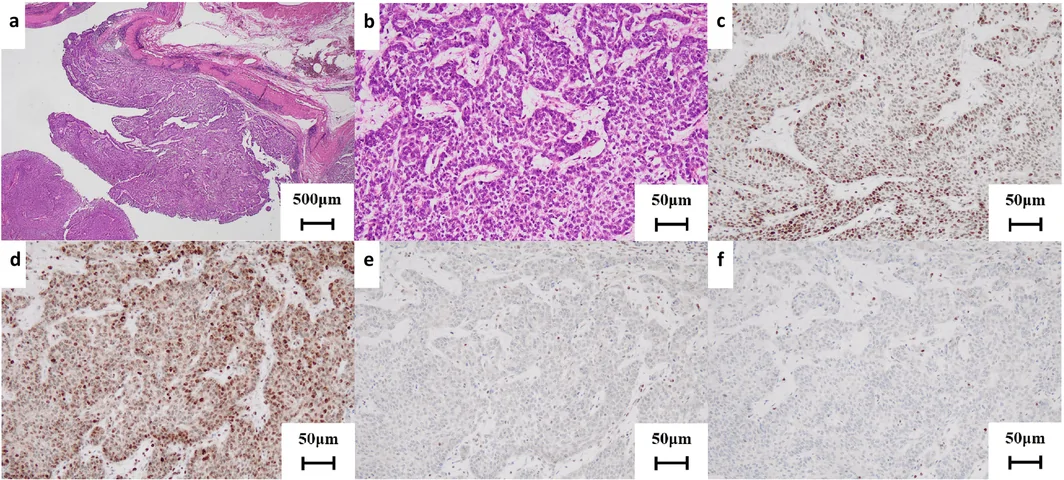
Histopathological and immunohistochemical findings of the resected renal pelvic tumor. (a) Low‐power view (hematoxylin and eosin staining [H&E], original magnification 20×), showing invasive urothelial carcinoma. (b) High‐power view (H&E, original magnification 200×) revealing atypical urothelial cells with enlarged nuclei and a high nuclear‐to‐cytoplasmic ratio, arranged in trabecular and nested patterns with infiltrative growth. (c–f) Mismatch repair immunohistochemistry showing nuclear expression of *MLH1* (c) and *PMS2* (d) was retained, whereas that of *MSH2* (e) and *MSH6* (f) was lost in tumor cells. The non‐neoplastic cells served as internal positive controls. These findings confirm deficient mismatch repair, consistent with the patient's known germline *MSH2* mutation.

Adjuvant gemcitabine and carboplatin chemotherapy was administered in two cycles. Follow‐up imaging 11 months postoperatively revealed enlargement of the right para‐aortic lymph node (12 mm) and increased fluorodeoxyglucose (FDG) uptake (Figure 3a). Computed tomography‐guided biopsy was technically unfeasible; therefore, endoscopic ultrasound‐guided biopsy through the small intestine was performed, although the obtained specimen was insufficient for pathological confirmation. Nodal recurrence of UTUC was strongly suspected based on the radiological findings and clinical course.

**FIGURE 3:**
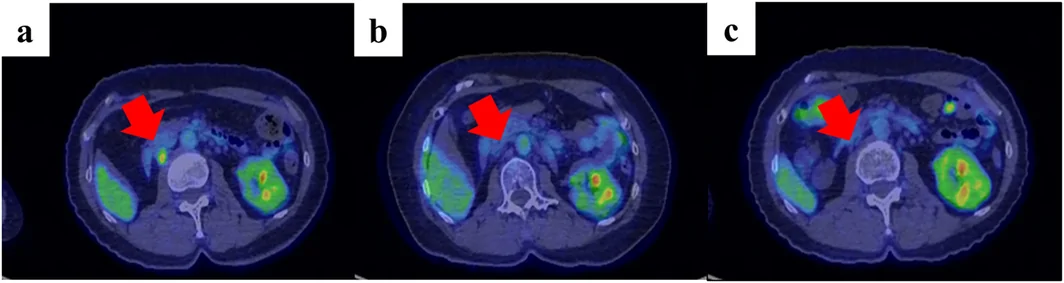
Chronologic imaging findings of postoperative para‐aortic lymph node recurrence and response to pembrolizumab. (a) Eleven months after radical nephroureterectomy, ^18^F‐fluorodeoxyglucose positron emission tomography/computed tomography (FDG‐PET/CT) demonstrated a right para‐aortic lymph node measuring 12 mm in short‐axis diameter with increased uptake (SUVmax 4.99), confirming nodal recurrence. (b) After 5 cycles of pembrolizumab, the lymph node decreased to < 10 mm on CT, and FDG‐PET/CT showed complete metabolic resolution, meeting the RECIST version 1.1 criteria for a complete response. (c) After 17 cycles of pembrolizumab, imaging confirmed sustained reduction of the lymph node and persistent absence of FDG uptake, confirming a durable complete response.

Pembrolizumab treatment was initiated at 200 mg every 3 weeks. After 5 cycles, the patient achieved a complete response (Figure 3b), as defined by the Response Evaluation Criteria in Solid Tumors (RECIST) version 1.1, with a reduction in the short‐axis diameter of the lymph nodes to ≤ 10 mm. Disappearance of metabolic activity was confirmed by positron emission tomography–computed tomography (PET/CT). Nevertheless, the patient developed an immune‐related adrenal insufficiency after 7 cycles, which was successfully managed with corticosteroid replacement therapy. At the time of writing, after more than 24 months without evidence of a new malignant tumor, the patient has maintained a complete radiological and metabolic response under continued treatment with pembrolizumab (Figure 3c).

## 3 Discussion

This case report describes a woman suffering from a genetically confirmed Lynch syndrome who developed postoperative nodal recurrence of renal pelvic carcinoma. Although pathological confirmation of the recurrence was not obtained due to insufficient biopsy samples, the imaging findings and clinical history strongly indicated nodal recurrence of UTUC. Treatment with pembrolizumab achieved a rapid and durable complete response, which has been maintained for ≥ 2 years.

Germline mutations in MMR genes cause Lynch syndrome, an inherited cancer predisposition syndrome associated with an increased risk for several malignancies, including UTUC [3, 5]. Specifically, MSH2 mutations confer a higher risk of UTUC [1, 4, 5]. Tumors that develop in this setting often exhibit MSI‐H status and dMMR [1, 2, 5]. As reported in several previous studies, MSI‐H UTUC is more often associated with underlying Lynch syndrome, although it is less common than in colorectal cancer [1, 2]. Latham et al. demonstrated that MSI‐H tumors across multiple cancer types, including urothelial tumors, were frequently associated with underlying Lynch syndrome [2]. Furthermore, carriers of pathogenic MSH2 variants are known to have a particularly high risk of UTUC development [4]. These findings suggest that MSI/MMR testing should be considered in patients with UTUC, particularly in those with early‐onset disease or multiple primary malignancies, because identification of Lynch syndrome has important implications for therapeutic decision‐making and genetic counseling [1, 2, 5].

The diagnosis of Lynch syndrome also has implications for the surgical management and postoperative surveillance of UTUC. Although kidney‐sparing treatment is an important option for selected patients with low‐risk UTUC, particularly when preservation of renal function is essential, radical nephroureterectomy remains the standard treatment for high‐risk UTUC. In the present patient, the lesion was considered clinically suspicious for invasive UTUC and the contralateral kidney was functional; therefore, radical nephroureterectomy with bladder cuff excision was selected with curative intent. The presence of Lynch syndrome alone does not necessarily mandate kidney‐sparing treatment. Nevertheless, patients with Lynch syndrome, particularly those with MSH2 mutations, remain at risk of developing metachronous contralateral UTUC. Accordingly, long‐term surveillance of the remaining upper urinary tract is particularly important.

MSI‐H/dMMR tumors are characterized by a high tumor mutational burden and increased neoantigen formation, which enhance tumor immunogenicity and sensitivity to immune checkpoint blockade [6, 9]. Pembrolizumab has demonstrated a clinical benefit in MSI‐H/dMMR solid tumors and advanced urothelial carcinoma, leading to durable responses [6, 7, 8]. In the present case, pembrolizumab was selected, given the patient's known dMMR status and suspected nodal recurrence following platinum‐based adjuvant therapy. Immune‐related endocrine adverse events are recognized complications of programmed cell death protein 1 inhibitors [10]. In this patient, adrenal insufficiency was successfully managed with corticosteroid replacement, allowing for the continuation of pembrolizumab treatment without loss of tumor control [10, 11, 12]. Recent data from Moussa et al. showed a confirmed response rate of 83%, including 16 complete responses among 24 patients with advanced dMMR/MSI‐H UTUC treated with single‐agent immune checkpoint inhibitors [13]. Cercek et al. [14] demonstrated the potential for organ preservation with neoadjuvant PD‐1 blockade in early‐stage dMMR solid tumors. Although the latter study addressed a different clinical setting, our case illustrates a durable response to pembrolizumab after postoperative nodal recurrence of Lynch syndrome‐associated UTUC and supports the clinical relevance of MMR testing for treatment selection.

In summary, this case highlights the potential role of immune checkpoint inhibition in MSI‐H/dMMR UC and underscores the importance of MSI testing in UTUC with a suspected hereditary background [1, 2, 5, 6].

## Funding

This study was supported by the Kochi University, Japan.

## Consent

Written informed consent was obtained from the patient.

## Conflicts of Interest

K.I. is a member of the Editorial Board of the *International Journal of Urology* and a co‐author of this article. To avoid any potential conflicts of interest, K.I. was excluded from all editorial decision‐making related to the acceptance of this manuscript.

## Data Availability

All data supporting the results of this study are available within the paper.

## References

[1] P. E. Gabriel , G. Cancel‐Tassin , F. Audenet , et al., “A Collaborative Review of the Microsatellite Instability/Deficient Mismatch Repair Phenotype in Patients With Upper Tract Urothelial Carcinoma,” BJU International 134 (2024): 723–735, 10.1111/bju.16405. 38813615

[2] A. Latham , P. Srinivasan , Y. Kemel , et al., “Microsatellite Instability Is Associated With the Presence of Lynch Syndrome Pan‐Cancer,” Journal of Clinical Oncology 37 (2019): 286–295, 10.1200/JCO.18.00283. PMC6553803 30376427

[3] X. Li , G. Liu , and W. Wu , “Recent Advances in Lynch Syndrome,” Experimental Hematology & Oncology 10 (2021): 37, 10.1186/s40164-021-00231-4. PMC8199357 34118983

[4] M. Dominguez‐Valentin , J. R. Sampson , T. T. Seppälä , et al., “Cancer Risks by Gene, Age, and Gender in 6350 Carriers of Pathogenic Mismatch Repair Variants: Findings From the Prospective Lynch Syndrome Database,” Genetics in Medicine 22 (2020): 15–25, 10.1038/s41436-019-0596-9. PMC7371626 31337882

[5] A. K. Lindner , G. Schachtner , G. Tulchiner , et al., “Lynch Syndrome: Its Impact on Urothelial Carcinoma,” International Journal of Molecular Sciences 22 (2021): 531, 10.3390/ijms22020531. PMC7825811 33430305

[6] A. Marabelle , D. T. Le , P. A. Ascierto , et al., “Efficacy of Pembrolizumab in Patients With Noncolorectal High Microsatellite Instability/Mismatch Repair‐Deficient Cancer: Results From the Phase II KEYNOTE‐158 Study,” Journal of Clinical Oncology 38 (2020): 1–10, 10.1200/JCO.19.02105. PMC8184060 31682550

[7] R. Oki , T. Urasaki , A. Ueki , et al., “A Radiological Complete Response to Pembrolizumab in a Patient With Metastatic Upper Urinary Tract Urothelial Cancer and Lynch Syndrome,” IJU Case Reports 6 (2023): 33–36, 10.1002/iju5.12542. PMC9807341 36605683

[8] K. Shiraishi , M. Shiota , G. Fukuchi , et al., “Complete Response to Pembrolizumab in Mismatch Repair‐Deficient Urothelial Carcinoma With Local Recurrence and Recurrent Upper Tract Urothelial Carcinoma: A Case Report,” International Cancer Conference Journal 14 (2025): 198–203, 10.1007/s13691-025-00774-x. PMC12229395 40625767

[9] D. M. O'Malley , G. M. Bariani , P. A. Cassier , et al., “Pembrolizumab in Microsatellite Instability‐High/Mismatch Repair Deficient (MSI‐H/dMMR) and Non‐MSI‐H/Non‐dMMR Advanced Endometrial Cancer: Phase 2 KEYNOTE‐158 Study Results,” Gynecologic Oncology 193 (2025): 130–135, 10.1016/j.ygyno.2024.12.020. 39847999

[10] P. Zhao , T. Zhao , L. Yu , W. Ma , W. Liu , and C. Zhang , “The Risk of Endocrine Immune‐Related Adverse Events Induced by PD‐1 Inhibitors in Cancer Patients: A Systematic Review and Meta‐Analysis,” Frontiers in Oncology 14 (2024): 1381250, 10.3389/fonc.2024.1381250. PMC11096456 38756658

[11] K. Sonehara , K. Tateishi , T. Hirabayashi , et al., “A Case of Lung Adenocarcinoma With Long‐Term Response After Late‐Onset Pembrolizumab‐Induced Acute Adrenal Insufficiency,” Case Reports in Oncology 14 (2021): 1–7, 10.1159/000508068. PMC7879310 33613234

[12] L. Escaño and P. Desai , “Secondary Adrenal Insufficiency as an Immune‐Related Adverse Event of Pembrolizumab Therapy,” Cureus 16 (2024): e65163, 10.7759/cureus.65163. PMC11339576 39176308

[13] M. J. Moussa , A. Y. Andreev‐Drakhlin , A. M. Venkatesan , et al., “Efficacy of Immune Checkpoint Blockade in Advanced Upper Tract Urothelial Cancer With DNA Mismatch Repair Deficiency or Microsatellite Instability,” JCO Precision Oncology 10 (2026): e2500772, 10.1200/PO-25-00772. PMC13048323 41911516

[14] A. Cercek , M. B. Foote , B. Rousseau , et al., “Nonoperative Management of Mismatch Repair‐Deficient Tumors,” New England Journal of Medicine 392 (2025): 2297–2308, 10.1056/NEJMoa2404512. PMC12661660 40293177

